# Examining the Factors Associated with Sustaining Jail-based Programs Linking to Community-based Medication for Opioid User Disorder (MOUD) Treatment

**DOI:** 10.21203/rs.3.rs-9419981/v1

**Published:** 2026-06-11

**Authors:** Margaret McGladrey, Michael Goetz, Julie Nakayima, Marisa Booty, Ashley Berkshire

**Affiliations:** University of Kentucky; University of Kentucky; University of Kentucky; University of Kentucky; Voices of Hope

**Keywords:** Opioid use disorder, implementation science, sustainment, linkage facilitation, jail healthcare, medications for opioid use disorder, reentry

## Abstract

Despite the prominence of sustainment as a construct in implementation science frameworks, there is limited literature on factors influencing the continued delivery and reach of evidence-based interventions in carceral settings after initial funding periods end. Extending previously published research on initial establishment of these programs with federally funded implementation support, this study reports on the post-intervention impact of jail-based programming to link people with opioid use disorder to medication treatment upon community reentry to assess whether and how linkage programming was maintained. In the five jails that sustained linkage program staff, only 4.9% of participants enrolled during the sustainment phase were successfully linked to community-based treatment, compared with 11.8% during the intervention phase. Interviews with linkage staff supervisors revealed that several factors salient to initial program implementation – adopter training and support, services and access, and systems and training – exerted ongoing effects during the sustainment period, demonstrating that preliminary implementation conditions may shape long-term reach. During the program sustainment period, these issues included restrictions on program eligibility criteria to reduce jail healthcare costs and deterioration of related clinical offerings (i.e., provision of in-house medication treatment). Additionally, challenges with maintaining data collection consistency after termination of the study’s data management infrastructure and reporting requirements resulted in significant missing data. Findings emphasize the need for enduring technical assistance and surveillance systems to support and monitor the impact of jail-based linkage programs as a component of the overall opioid use disorder continuum of care.

## Introduction

Jail settings are uniquely positioned to reduce the high risk of opioid overdose at community reentry, but their complex dual public safety and public health functions entail significant investments of time and resources to implement evidence-based overdose reduction practices. As such, there is a need for implementation science research on factors associated with the integration and sustainment of jail-based linkage programs after the intervention period and research infrastructure support have ended (Grella et al., 2022). The HEALing Communities Study (HCS) was a cluster randomized waitlist-control intervention that sought to reduce opioid overdose deaths in four states (Kentucky, Massachusetts, New York, Ohio) via implementation of overdose education and naloxone distribution and medication for opioid use disorder (MOUD) programs (Davis et al., 2024; Walsh et al., 2020). The HCS-Kentucky (HCS-KY) site included eight counties during the Wave 1 intervention period (January 2020 – June 2022) and eight counties during the Wave 2 waitlist-control period (July 2022 – December 2023) (Davis et al., 2024). A previous *Health & Justice* manuscript about the Wave 1 jail-based linkage programs using the Practical Robust Implementation and Sustainability Model (PRISM) found that initial sustainment decisions were not associated with program reach (operationalized in this study as the number of participants enrolled, number of visits, number of participants referred to MOUD for each jail site) or by rural/urban location (McGladrey, et al., 2025). Therefore, further research is needed to understand factors influencing longer-term jail-based linkage program sustainment.

This article examines implementation factors impacting the reach of sustained jail-based linkage programs after the conclusion of the Wave 1 and 2 periods. In this brief report, we provide updated reach data for jail-based linkage programs for the time period immediately following HCS (30 months for programs implemented during Wave 1 and 18 months for Wave 2) to assess whether reach was maintained, expanded, or diminished following the conclusion of HCS-KY support. We also include interview data from VOH linkage program supervisors to examine the potential influence of PRISM factors that shaped initial implementation on sustained programming.

## Methods

### Original intervention

The jail-based linkage program was designed to address gaps in MOUD access among people detained in jails and transitioning back to the community. During HCS-KY, two community-based organizations were contracted to deploy and manage linkage workforces embedded within local jails to provide individualized, pre- and post-release navigation to facilitate MOUD initiation and continuity of care at reentry (see McGladrey, et al., 2025 for linkage program component details). HCS-KY implementation facilitators partnered with jail leadership to align program operations with facility practices, assess and match staffing capacity to expected service volume, and support linkage staff onboarding through guided planning and transition activities (McGladrey, et al., 2025).

To support workforce readiness and fidelity, HCS-KY developed and delivered specialized training programs for MOUD linkage staff in the absence of existing comprehensive curricula (McGladrey, et al., 2025). Ongoing implementation support included monthly cross-site meetings, development of agency-specific standard operating procedures, and site-level implementation plans to facilitate integration within jail contexts (Hogue et al., 2024; McGladrey, et al., 2025). These supports were modified but not eliminated following the conclusion of HCS-KY, as sustainment planning emphasized continuity of core functions alongside adaptation to new funding and oversight structures.

Beginning in spring 2022, implementation facilitators worked with jail liaisons to plan for sustainment by reviewing program reach data, staffing models, projected costs, and potential funding mechanisms, including opioid settlement resources (McGladrey, et al., 2025). Voices of Hope (VOH) is the focus of the present analysis because only the VOH-staffed linkage programs were sustained post-intervention due to changes in liability insurance for the second linkage staff provider. VOH secured state funding from the Kentucky Opioid Response Effort (supported by the SAMHSA State Opioid Response block grant) and a grant from the Kentucky Opioid Abatement Advisory Commission to maintain linkage positions in interested jails. Transition meetings facilitated hand-offs from HCS-KY oversight to VOH leadership, preserving core linkage activities while shifting supervisory and administrative responsibility.

### IRB and data collection

The protocol (Pro00038088) was approved by Advarra Inc., HCS’s single Institutional Review Board, and was granted an addendum for HCS-KY to receive additional data from VOH for jails participating in sustainment in both the Wave 1 and Wave 2 communities.

### Data sources

A Research Electronic Data Capture (REDCap v14.4.1) system was developed by HCS and utilized to collect data during the implementation period for both waves of the study. During the sustainment period, client data were inputted into the RecoveryLink system maintained by VOH. The input data were then de-identified and aggregated by VOH and transferred to HCS. These data included the number of participants enrolled, coaching sessions, participants linked to MOUD, and demographics. Data examining the reach of the linkage program during the intervention and sustainment periods were summarized from REDCap and VOH’s reports, respectively.

Additionally, the fifth author, who serves as VOH’s data manager, conducted brief interviews in late 2025 with VOH supervisors of jail-based linkage staff about implementation status by asking what barriers and facilitators they encountered in delivering linkage programming at each jail site.

### Analysis

Among the 8 communities randomized to the Wave 1 period, 5 selected MOUD linkage in jails as a strategy; of those, 3 participated in sustainment. In the Wave 2 period, 5 of the 8 communities participated in the intervention strategy, and 2 sustained those efforts. This descriptive analysis focuses on the 5 sustaining communities to assess the factors associated with jails’ maintenance of MOUD linkage programs.

## Results

### Reach of Sustained Linkage Programs

The initial linkage programming implementation phase ranged from 7.5 to 12 months for Wave 1 jails and from 8.5 to 10 months for Wave 2 jails. Following this period, the sustainment period lasted 24 months for Wave 1 jails and 12 months for Wave 2 jails. Table 1 displays the implementation and sustainment timelines, program participation, and reach for each jail. Of the five jails engaged in sustainment efforts, three (60%) were located in rural areas. The reach of jails within the intervention group was discussed previously among Jails A, C, and E, which also participated in sustainment; these previously assigned letters were maintained in this paper (McGladrey et al., 2025).

Across the Wave 2 jails (F and G), 45 individuals enrolled in the linkage program during the intervention period, with a total of 247 visits with linkage staff (an average of 5 visits per participant), and 40 were referred to MOUD at one of these visits. Ten participants were confirmed to have entered MOUD treatment post-release.

A total of 365 participants were enrolled in the sustainment phase, with the majority (310; 85%) originating from the Wave 1 jails. These 310 participants from the Wave 1 group completed 2,406 coaching sessions, and 18 individuals (6%) were successfully linked to MOUD. In contrast, the 55 participants from the Wave 2 jails completed 312 coaching sessions, with no reported linkages to MOUD. Overall, 18 of the 365 participants (4.9%) in the sustainability phase were linked to MOUD, compared to 25 of the 212 participants (11.8%) during the intervention phase.

### Characteristics of Linkage Program Participants

Table 2 displays the characteristics of 365 participants using data from RecoveryLink. It is important to note the enormity of missing data, ranging from 2.7% to 52.9%, due to VOH’s transition from REDCap to RecoveryLink, which does not have a forced response requiring linkage staff to submit answers.

Jails C (41.2%), E (52.8%), and F (42.3%) enrolled higher percentages of females, compared to jails A (52.4%) and G (34.5%), which enrolled more males. Most participants enrolled in jails A (90.3%), C (47.1%), E (82.4%), and F (76.9%) were white, compared to a near-majority in jail G (48.3%). More non-Hispanic participants were also enrolled, except for Jail G.

### Influence of PRISM Factors on Sustained Linkage Programs

As in the original article, we stratify sites as having high or low reach based on their relative numbers of participant enrollments, visits, and confirmed linkage to MOUD (Jails A and E are high-reach, Jails B, C, and D are low-reach). Interviews with VOH supervisors of jail-based linkage staff indicated that several PRISM factors shaping initial implementation continued to distinguish high-reach from low-reach linkage program sites. Relevant to the *systems and training* PRISM factor (i.e., jail infrastructure supportive of implementation), three jails experienced changes to MOUD availability during the transition from intervention to sustainment. In Jail A, the naltrexone provider was no longer in place to offer pre-release injections during the first year of sustainment, leading to treatment disruptions that were not remedied until the beginning of 2025. Yet, linkage program enrollments remained steady because the facility only offered naltrexone, so the need for linkage to buprenorphine and methadone continued. In the lower-reach Jail F, MOUD is now offered in the jail, but linkage staff may not have counted MOUD initiation as linkage program participation while VOH reclassified the program as “retention” rather than linkage. The HCS intervention successfully expanded access to long-acting injectable buprenorphine in Jail G, but the county fiscal court refused to continue paying MOUD treatment invoices after the study ended, which closed the most efficient linkage pathway for this lower-reach program.

Relatedly, the *services and access* PRISM factor (i.e., referral pathways for identifying and engaging with incarcerated clients) exerted continued influence on linkage programming sustainment as jail sites tightened eligibility criteria to control in-house MOUD costs. For example, at lower-reach Jail G, despite the jailer’s support for MOUD access, the lack of fiscal court funding during sustainment led the jail to focus on “short-timers” who would likely be released within 30–60 days. Lower-reach Jail C placed similar priority on clients nearing release, which the linkage supervisor saw as a limit on support that could begin at the time of incarceration. Additionally, Jail C does not allow linkage staff access to laptops within the facility, necessitating paper documentation that undermines efficiency and data security in the fast-paced jail setting. They are also the only facility requiring NCIC-level background checks, a *legal factor* that slows staff recruitment and training. By contrast, the higher-reach Jail E has emphasized screening all residents within 48 hours of intake, especially as many residents will be quickly released. Jail E affords linkage staff the ability to move independently through the jail without competing for meeting rooms with lawyer visits and other programming, and overdose education and SMART meetings at have also offered opportunities to recruit residents.

The stark difference in the overall reach of the implementation and sustainment phase programs – particularly of the Wave 2 programs from which only 15% of sustainment phase enrollments originated – indicates the influence of the *adopter training and support* PRISM factor related to HCS training and supporting linkage staff. From initial implementation of the intervention to Wave 2 programming and eventual sustainment efforts, diminished adopter training and support from HCS over time seemed to undermine program reach, or at least our ability to assess it due to discontinuation of HCS data management infrastructure post-intervention.

## Discussion

This report highlights the implementation factors impacting the reach of sustained jail-based MOUD linkage programs after the conclusion of the HCS-KY Wave 1 and 2 interventions. Sites with higher reach during the HCS implementation period continued to demonstrate higher reach during sustainment, suggesting that early system readiness and implementation quality may have durable effects. Erosion of *adopter training and support* during sustainment may weaken programs that initially depended on structured implementation facilitation. Research should further examine how PRISM contextual and infrastructure factors influence long-term performance beyond the active implementation phase.

Additionally, services and access acted as a barrier in facilities where linkage staff had limited ability to connect with the broader jail population, while expanded linkage staff access to potential clients resulted in higher reach. In this way, referral pathways and eligibility decisions can constrain population impact. Some jails narrowed eligibility for linkage services during sustainment to manage costs, which may have limited reach. Practitioners and policymakers should consider how referral criteria and access rules affect program goals and equity, particularly in resource-constrained settings.

Findings suggest that sustaining linkage programming requires preserving clinical and system supports, not only staffing. Loss of MOUD availability and pre-release medication disrupted continuity of care in some sites, even when linkage staff positions were sustained, which underscores the importance of aligning staffing, medication access, and clinical workflows when planning for sustainment.

Finally, this report highlights that measurement consistency is essential for interpreting sustainment outcomes. Variation in how “linkage” was defined, especially in sites with in-house MOUD, complicated comparisons across jails and over time. Future research should prioritize consistent terminology, aligned data systems, and clear operational definitions to strengthen reach assessment and support more rigorous sustainment evaluations. Given these measurement and implementation challenges, it is unsurprising that only a small proportion of incarcerated individuals who enrolled in linkage programs initiated MOUD. Jail-based linkage programs should be evaluated through public surveillance systems as one component of a broader system of care rather than as a standalone research intervention.

## Figures and Tables

**Table 1:** Linkage Program Implementation and Sustainment Timelines and Reach

Jail Site*	Jail A	Jail C	Jail E	Jail F	Jail G
Intervention wave	1	1	1	2	2
Urbanicity of jail location’s county	Urban	Urban	Rural	Rural	Rural
Implementation Program Timeline					
Intervention period	Jul 2021 to Dec 2022			Jan 2023 to Dec 2023	
First participant enrollment date	12/14/21	2/28/22	7/8/21	2/21/23	4/10/23
Total # implementation months	7.5 months	7.5 months	12 months	10 months	8.5 months
Implementation Program Reach					
Total # participant enrollments, [n]	46	14	107	20	25
Total # participant visits, [n]	193	23	377	132	115
Total # ever referred to MOUD at any visit, [n]	39	10	23	17	23
Total # confirmed to have entered MOUD, [n]	8	1	6	7	3
Sustainment Program Timeline and Reach					
Sustainment period	Jan 2023 to Dec 2024			Jan 2024 to Dec 2024	
Total # participant enrollments, [n]	185	17	108	26	29
Total # linked to MOUD, [n]	4	0	14	0	0
Coaching sessions, [n]	1,496	23	887	210	102

* Jail site was redacted for confidentiality reasons

**Table 2:** Demographic Characteristics of Sustained Linkage Programs

Jail Site*	Jail A	Jail C	Jail E	Jail F	Jail G
Total enrolled, [n]	185	17	108	26	29
Demographics					
Gender [n (%)]					
Female	76 (41.1)	7 (41.2)	57 (52.8)	11 (42.3)	4 (13.8)
Male	97 (52.4)	1 (5.9)	34 (31.5)	9 (34.6)	10 (34.5)
Unknown or missing	12 (6.5)	9 (52.9)	17 (15.7)	6 (23.1)	15 (51.7)
Ethnicity [n (%)]					
Hispanic	1 (< 1)	1 (5.9)	2 (1.9)	0 (0.0)	0 (0.0)
Non-Hispanic	180 (97.3)	9 (52.9)	82 (75.9)	20 (76.9)	9 (31.0)
Unknown or missing	4 (2.2)	7 (41.2)	24 (22.2)	6 (23.1)	20 (69.0)
Race [n (%)]					
White	167 (90.3)	8 (47.1)	89 (82.4)	20 (76.9)	14 (48.3)
Black	9 (4.9)	1 (5.9)	2 (1.9)	0 (0.0)	0 (0.0)
More than 1 race	4 (2.2)	1 (5.9)	3 (2.8)	0 (0.0)	0 (0.0)
Unknown or missing	5 (2.7)	7 (41.2)	14 (13.0)	6 (23.1)	15 (51.7)

* Jail site was redacted for confidentiality reasons

There is a substantial amount of missing demographic information because the data collection instruments (Recovery link and Recovery Data Platform) lack forced-response programming options.

## Data Availability

The de-identified datasets used in the current study are available from the corresponding author on reasonable request.

## Abbreviations

HCS: HEALing Communities Study
HCS-KY: HEALing Communities Study-Kentucky
MOUD: Medications for opioid use disorder
PRISM: Practical Robust Implementation and Sustainability Model
VOH: Voices of Hope
